# Diagnostic delay of autism in Jordan: review of 84 cases

**DOI:** 10.3402/ljm.v8i0.21725

**Published:** 2013-08-19

**Authors:** Amira T. Masri, Najati Al Suluh, Ramzi Nasir

**Affiliations:** 1 Division of Child Neurology, Department of Pediatrics, Faculty of Medicine, University of Jordan, Amman, Jordan; 2 Department of Developmental Behavioral Pediatrics, Boston Children's Hospital, Harvard Medical School, Boston, MA, USA

Little research is available on autism spectrum disorders (ASDs) epidemiology and clinical practice in developing countries. Studies from the Middle East are particularly rare (1–6). In Western countries, autism is regarded as highly influenced by genetics, although genetic abnormalities are only identified in a minority of patients (7). There is increasing evidence that the high rates of consanguinity in the Middle East predispose to an autosomal recessive pattern of inheritance of autism (8). In Jordan, the incidence of autosomal recessive disorders is high, and contributes significantly to the etiologies of global developmental delay (9, 10). However, it is not known if autosomal recessive disorders contribute to the incidence of autism in Jordan.

In this study, we reviewed the medical records of all the 84 children (64 boys and 20 girls) diagnosed with autism between January 2001 and December 2009, at our clinic at Jordan University Hospital. The Clinical diagnosis of autism was based on the DSM: IV_TR criteria, American Psychiatric Association (11).

## Patients’ characteristics

### Age of onset of symptoms and age of presentation to clinic

Regarding the patients’ characteristics, in 46 (54.7%) patients, parents noticed the delay without history of a period of regression, while regression was noticed in 38 (45.2%) patients. The age of onset of symptoms in the regressive group ranged from 1 to 30 months with an average age of 15 months. The age of presentation to our clinic and the diagnosis of autism ranged from 14 months to 9 years, with an average age at diagnosis of 3.8 years.

The most common cause for presentation to the clinic was speech delay: 41 patients (48.8%). Associated symptoms in addition to the social delay included stereotyped movements (70.2%), hyperactivity (61.9%), agitation (27.4%), and motor delay (15.5%).

### Risk factors

Regarding risk factors, neither the antenatal nor the birth history proved to be risk factor. Furthermore, family history of consanguinity was reported in 38 (45.2%) patients, family history of autism was reported in seven (8.3%) patients, and family history of global developmental delay was reported in 19 (22.6%) patients.

### Follow-up and investigations

The total period of follow-up ranged from a few months to 3 years. Investigations including brain MRI, chromosomal analysis standard and fragile X, and phenylalanine level were requested in all of the patients. Many parents (28 patients, 33.3%) did not continue the work-up. All of the work-up done was normal apart from fragile X testing, which was abnormal in two patients (4.7%), and phenylalanine level, which was abnormal in two patients (4.3%).

### Parent knowledge regarding autism diagnosis

Although all parents were aware that their children had some delay before the age of 30 months, only very few (7%) were aware that their child might have autism. Only three (3.6%) patients were diagnosed as having autism before presenting to us, four (4.7%) were referred by pediatricians for suspicion of autism, and 11 (13.1%) were attending rehabilitation centers.

We noticed that despite early parent concerns regarding their child's development, only a minority of patients had early diagnosis or referral. This delay most likely reflects the lack of screening for autistic spectrum disorders by pediatricians in our country. More education for pediatricians about autism symptoms and tools for screening might have a great impact on early referral and outcome in our country. Furthermore, a minority of parents had specific concerns about autism indicating the need for increased awareness among the public.

We were not able to identify an underlying cause in most of the patients, consistent with previous studies that revealed an identifiable cause in ≤10% of patients (7). In our study, an underlying cause for autism was found in only four (5%) patients. It is of interest to note that the etiologic yield in our earlier study on children with global developmental delay was 44.5% (9), compared to 5% in this study on autism. We also note that in this setting, more expensive diagnostic tests are not available such as Chromosomal Microarrays, Molecular testing for Fragile X, and sequencing of genes associated with Rett Syndrome.

The high rate of consanguinity in this cohort suggests the potential for the presence of rare and yet undiscovered autosomal recessive disorders in autism, as recently described in similar populations in the Middle East (8). It is also possible that in a subset of these patients, the etiology of autism is multifactorial with a variety of genetic and, to a lesser extent, environmental factors playing a role (12). An important limitation to our study is the limited number of patients who had diagnostic evaluation.

In conclusion, autism needs more public and health care provider awareness in Jordan. The high rate of consanguinity in families with autistic children suggests the possibility of yet undiscovered autosomal recessive contributions.

*Amira T. Masri* Division of Child Neurology Department of Pediatrics, Faculty of Medicine University of Jordan Amman, Jordan Email: masriamira69@hotmail.com *Najati Al Suluh* Division of Child Neurology Department of Pediatrics, Faculty of Medicine University of Jordan Amman, Jordan *Ramzi Nasir* Department of Developmental Behavioral Pediatrics Boston Children's Hospital Harvard Medical School Boston, MA, USA
